# Phenotypic Biodiversity and Niche-Associated Functional Traits in *Lactiplantibacillus plantarum*

**DOI:** 10.3390/cimb48070683

**Published:** 2026-07-02

**Authors:** Gianluca Paventi, Mariantonietta Succi, Katia Maglieri, Catello Di Martino, Maria Virginia Soldovieri, Massimo Iorizzo

**Affiliations:** 1 Department of Agriculture, Food, Natural Resources and Engineering (DAFNE), University of Foggia, Via Napoli, 25, 71122 Foggia, Italy; 2 Department of Agricultural, Environmental and Food Sciences, University of Molise, Via De Sanctis, 86100 Campobasso, Italy; succi@unimol.it (M.S.); lello.dimartino@unimol.it (C.D.M.); 3 Department of Medicine and Health Sciences “V. Tiberio”, University of Molise, Via De Sanctis, 86100 Campobasso, Italy; katia.maglieri@studenti.unimol.it (K.M.); mariavirginia.soldovieri@unimol.it (M.V.S.)

**Keywords:** lactic acid bacteria, strain variability, ecological origin, carbohydrate metabolism, β-glucosidase activity, multivariate analysis, PERMANOVA

## Abstract

*Lactiplantibacillus plantarum* is a highly versatile lactic acid bacterium, widely distributed across diverse ecological niches. Although often described as a nomadic species, increasing evidence suggests that strains from different habitats may retain niche-associated functional traits. This study investigated the phenotypic biodiversity of forty *L. plantarum* strains isolated from four ecologically distinct environments: wine, honeybee gut, trout intestine, and pre-weaning infant feces. Growth performance at different temperatures and on various carbon sources, acidification capacity, and β-glucosidase activity were evaluated and integrated using multivariate statistical analyses. Significant differences in β-glucosidase activity were observed among ecological groups (Kruskal–Wallis, *p* = 0.001), with wine-associated strains exhibiting the highest enzymatic activities and trout-derived isolates the lowest. Growth and acidification traits showed more limited variation among habitats, indicating that these physiological characteristics are largely conserved within the species. Heatmap visualization, principal component analysis (PCA), and hierarchical clustering revealed substantial phenotypic heterogeneity among strains. PCA indicated that growth performance and acidification traits contributed primarily to the first principal component, whereas β-glucosidase activity and differential fructose utilization were major contributors to the second component. Permutational multivariate analysis of variance (PERMANOVA) confirmed a significant effect of ecological origin on the overall phenotypic structure (*p* = 0.006), although habitat explained only 15.3% of the total variance (R^2^ = 0.153). Overall, the results show that ecological origin contributes to the phenotypic diversification of *L. plantarum* populations while preserving the extensive functional versatility characteristic of this species. β-Glucosidase activity emerged as the most discriminating phenotypic trait among ecological groups and represented the principal niche-associated functional signature identified in this study.

## 1. Introduction

*Lactiplantibacillus plantarum* (formerly *Lactobacillus plantarum*) is one of the most versatile and ecologically widespread species among lactic acid bacteria (LAB) [1]. Owing to its remarkable metabolic flexibility, it has been isolated from a broad range of habitats, including fermented vegetables, cereals, fruits, wine, dairy products, insects, and the gastrointestinal tracts of humans and animals [1,2]. This extensive ecological distribution, together with its technological relevance in food fermentations and its probiotic potential, has made *L. plantarum* one of the most intensively studied LAB species [3,4].

The ecological success of *L. plantarum* is closely linked to its exceptional genomic plasticity. Recent genomic investigations have also highlighted the remarkable metabolic adaptability of food-associated *L. plantarum* strains, demonstrating extensive variability in carbohydrate utilization pathways, stress-response systems, and probiotic-related functions [5].

Comparative genomic studies have revealed an open pangenome composed of a relatively conserved core genome and a large accessory genome enriched in genes involved in carbohydrate metabolism, environmental adaptation, stress responses, and host interactions [6,7]. Recent pan-genomic analyses have further highlighted the remarkable evolutionary potential of the species, supporting its ability to colonize highly diverse ecological niches [3,8]. The presence of plasmids, bacteriophage-associated regions, mobile genetic elements, and CRISPR-Cas systems further contributes to strain-specific diversification and adaptive potential. Recent large-scale genomic analyses have further demonstrated that, despite the widespread conservation of key metabolic functions, *L. plantarum* populations exhibit substantial strain-level functional diversity, reinforcing the view that intraspecific variability represents a major determinant of ecological versatility and biotechnological potential [9].

Because of these characteristics, *L. plantarum* has frequently been described as a “nomadic” or “generalist” species capable of transiently colonizing multiple habitats without exhibiting strong habitat-specific genomic signatures [7,10,11]. Large-scale comparative genomic analyses have often revealed weak correlations between phylogeny and habitat of origin, suggesting that broad metabolic versatility rather than strict ecological specialization underlies the evolutionary success of the species.

Nevertheless, increasing evidence indicates that strains originating from different ecological environments may retain measurable functional signatures associated with their habitat of isolation. Several studies have reported ecological origin-dependent differences in carbohydrate utilization, stress tolerance, antimicrobial activity, carbohydrate-active enzyme repertoires, and probiotic-related properties [12,13,14,15,16]. For example, a study demonstrated that some *L. plantarum* strains isolated from different fermented foods exhibited distinct metabolic and stress-resistance phenotypes, reflecting adaptation to specific ecological conditions [14,17,18,19]. Similarly, other studies reported niche-dependent differences in carbohydrate-active enzyme profiles and carbohydrate utilization capabilities, supporting the existence of habitat-associated functional diversification [13,20]. These observations suggest that ecological adaptation in *L. plantarum* may occur through the differential distribution of functional traits rather than through the emergence of strictly habitat-specific lineages.

The ecological niches considered in the present study represent markedly different physicochemical environments. Wine constitutes a highly selective habitat characterized by low pH, ethanol, organic acids, sulfites, and grape-derived phenolic compounds. In contrast, the honeybee gut is associated with nectar- and pollen-derived substrates rich in simple sugars, flavonoids, and plant secondary metabolites and hosts a specialized microbial community involved in nutrition and host health [21]. The trout gastrointestinal tract is characterized by lower environmental temperatures and dietary conditions associated with aquatic ecosystems, whereas the intestinal environment of pre-weaning infants is shaped by milk-derived nutrients, host developmental processes, and the progressive establishment of the gut microbiota. Such contrasting ecological conditions may impose selective pressures capable of influencing the distribution of functional traits within the same bacterial species.

Among the phenotypic characteristics potentially involved in ecological adaptation, carbohydrate metabolism and glycosidase activities are of particular interest. β-Glucosidases are among the most relevant enzymes in food fermentation because they catalyze the hydrolysis of glycosidic bonds, promoting the release of bioactive aglycones and aroma-active compounds and thereby improving the nutritional, sensory, and functional quality of fermented products [22,23]. In *L. plantarum*, β-glucosidase activity has attracted considerable attention because of both its ecological significance and its technological potential [24]. Several studies have reported substantial strain-dependent variability in β-glucosidase activity, suggesting that ecological origin may influence enzyme expression and functionality [15,18,19]. Furthermore, previous investigations have documented remarkable biodiversity among wine-associated *L. plantarum* populations [25], supporting the use of β-glucosidase activity as a model functional trait for exploring habitat-associated phenotypic diversification.

Because the ecological niches investigated in the present study differ markedly in substrate composition and physicochemical characteristics, β-glucosidase activity was selected as a key functional marker to explore the relationship between habitat, phenotypic diversity, and adaptive potential within the species. Growth performance under different environmental conditions and acidification capacity were also evaluated as complementary indicators of metabolic competence and ecological efficiency.

Despite the growing body of evidence supporting functional diversification within *L. plantarum*, relatively few studies have simultaneously compared strains isolated from ecologically distant habitats using an integrated phenotypic approach. In particular, comparative investigations involving strains originating from wine, honeybee-associated environments, fish gastrointestinal tracts, and the infant gut remain scarce. Understanding whether strains isolated from these contrasting niches retain distinct functional signatures is essential for elucidating the ecological mechanisms underlying intraspecific diversification and for identifying strains with potential technological, probiotic, and biotechnological applications.

Therefore, the aim of the present study was to investigate the phenotypic biodiversity of forty *L. plantarum* strains isolated from four ecologically distinct niches—wine, honeybee gut, trout intestine, and infant feces. By combining enzymatic, physiological, and metabolic characterization with multivariate statistical analyses, we sought to determine whether ecological origin is associated with measurable functional signatures and to evaluate the contribution of niche-related adaptation to the diversification of this highly versatile bacterial species.

## 2. Materials and Methods

### 2.1. Bacterial Strains and Culture Conditions

The study included 40 *L. plantarum* isolates obtained from four ecologically distinct niches: honeybee gut (n = 10), wine (n = 10), pre-weaning infant feces (n = 10), and trout intestine (n = 10). To capture diversity within each ecological group, isolates were recovered from multiple biological sources, including five different red wines, six wild trout specimens, six *Apis mellifera* colonies, and three healthy pre-weaning infants. Multiple isolates were obtained from some samples and subsequently maintained as independent strains in the microbial culture collection of the Department of Agricultural, Environmental and Food Sciences (DiAAA), University of Molise (Campobasso, Italy).

All isolates had been previously identified as *Lactiplantibacillus plantarum* by 16S rRNA gene sequencing. The corresponding GenBank accession numbers, together with strain designations, ecological origin, and year of isolation, are reported in Supplementary Table S1.

Wine-associated isolates were obtained from traditional Italian wines [25], honeybee isolates from *Apis mellifera ligustica* colonies [26], and trout isolates from the intestinal tract of Mediterranean trout (*Salmo macrostigma*) [27]. Infant-derived isolates originated from fecal samples collected from healthy pre-weaning infants within a research project approved by the Bioethical Committee of the University of Molise (Protocol No. 21779, 9 May 2024). The results of this project have not been previously published. All strains were stored at −80 °C in MRS broth supplemented with glycerol until use.

Prior to experimentation, strains were routinely propagated in de Man–Rogosa–Sharpe (MRS) broth (Oxoid, Basingstoke, UK) and incubated under anaerobic conditions. Anaerobic conditions were generated using AnaeroGen™ sachets (Oxoid, UK) in sealed anaerobic jars. All strains were revived and propagated under identical culture conditions prior to phenotypic characterization in order to minimize procedural variability among isolates. Cell suspensions were standardized to 0.5 McFarland. Preliminary viable counts performed on MRS agar confirmed that this turbidity corresponded approximately to 10^8^ CFU mL^−1^ under the experimental conditions employed. These standardized suspensions were then used as inocula for all subsequent assays. All experiments were performed using three independent biological replicates.

### 2.2. Growth Characterization Under Different Environmental Conditions

Growth performance was evaluated in MRS broth at three incubation temperatures (22, 28, and 37 °C). The selected temperatures were chosen to cover the mesophilic growth range typically associated with *L. plantarum* [28]. The three temperatures were initially evaluated to identify the condition supporting the highest overall growth performance. Based on these preliminary results, 37 °C was selected for all subsequent physiological and functional assays, as it represented the optimal growth temperature for the majority of the strains.

Cultures were inoculated to obtain an initial optical density (OD_600_) of approximately 0.1 and incubated under anaerobic conditions. Bacterial growth was monitored spectrophotometrically by measuring optical density at 600 nm (OD_600_) using a Basic Eppendorf spectrophotometer (Eppendorf SE, Hamburg, Germany).

To evaluate the effect of the carbon source, additional growth assays were performed at 37 °C in modified MRS broth in which glucose was replaced by fructose (20 g L^−1^). Cultures were incubated for 72 h under anaerobic conditions.

### 2.3. Acidification Assays

The acidification capacity of each strain was evaluated in MRS broth containing either glucose or fructose as the principal carbohydrate source. Cultures were incubated anaerobically at 37 °C, and pH values were recorded at 0, 6, 24, 48, and 72 h. Final pH values recorded after 72 h were used for comparative phenotypic analyses and multivariate statistical evaluations.

### 2.4. β-Glucosidase Activity Assay

β-Glucosidase activity was determined according to the method of Ávila et al. [29], with minor modifications. The cultivation medium used prior to the enzymatic assay was maintained substantially consistent with the original protocol to allow comparison with previously reported β-glucosidase activity data in LAB. Cells were harvested after overnight incubation, corresponding to the late exponential/early stationary growth phase under the cultivation conditions employed. The glucose-free medium was used to minimize catabolite repression effects and to avoid possible inhibition of β-glucosidase expression by readily metabolizable carbohydrates. This approach is consistent with previous studies evaluating β-glucosidase activity in lactic acid bacteria and facilitates comparison with published data obtained using similar experimental conditions.

Strains were cultivated overnight in a modified MRS medium lacking meat extract and glucose and supplemented with lactose (0.5 g L^−1^), Tween 80 (0.2 g L^−1^), acid casein hydrolysate (0.8 g L^−1^), and cysteine (0.05 g L^−1^). Cultures (10 mL) were centrifuged at 8000 rpm for 10 min at 4 °C, washed twice with sterile saline solution (0.9% NaCl, *w*/*v*), and resuspended in phosphate-buffered saline (PBS, pH 7.4).

Cell suspensions were standardized to an OD_600_ of 0.5. The enzymatic reaction mixture consisted of 50 μL of 20 mM p-nitrophenyl-β-D-glucopyranoside (p-NPG), prepared in PBS (pH 6.5), and 50 μL of bacterial suspension, adjusted to a final volume of 1 mL with PBS. Aliquots (200 μL) were transferred into sterile 96-well microplates and incubated in a Multiskan™ FC microplate reader (Thermo Fisher Scientific, Waltham, MA, USA). Absorbance was monitored kinetically at 405 nm for 6 h. β-Glucosidase activity was expressed as nmol of p-nitrophenol (pNP) released per milliliter of reaction mixture at the end of the experimental test (nmol pNP mL^−1^ released in 6 h). Quantification of pNP was performed using a calibration curve prepared with standard p-nitrophenol solutions. All assays were performed in triplicate. Because intact cell suspensions were used in the assay without prior cell disruption, the measured activity should be interpreted as cell-associated (whole-cell) β-glucosidase activity rather than extracellular or total enzymatic activity.

### 2.5. Statistical and Multivariate Analyses

All experiments were performed using three independent biological replicates. Each biological replicate was analyzed in technical duplicate, and the mean value of the technical replicates was used for subsequent statistical analyses. Differences in β-glucosidase activity among ecological groups were evaluated using the Kruskal–Wallis test. When significant differences were detected, pairwise comparisons were performed using Dunn’s post hoc test with Holm correction for multiple testing, and the results are reported in Supplementary Table S3. Prior to PCA, heatmap construction and hierarchical clustering, phenotypic variables were standardized using Z-score transformation. Principal component analysis (PCA) was then performed using the standardized dataset, including β-glucosidase activity, growth performance under different temperature and carbon-source conditions, and acidification traits. Hierarchical clustering was conducted using Euclidean distance and Ward’s linkage method. The influence of ecological origin on the overall phenotypic structure was assessed by permutational multivariate analysis of variance (PERMANOVA) performed on a Euclidean distance matrix calculated from the standardized phenotypic dataset using 999 permutations. Homogeneity of multivariate dispersions (PERMDISP) was also evaluated to assess differences in within-group dispersion. Statistical significance was accepted at *p* < 0.05. All statistical analyses and graphical representations were performed using Python (version 3.12) with the packages pandas, NumPy, SciPy, scikit-learn, seaborn and matplotlib. The complete raw phenotypic dataset used for heatmap construction, principal component analysis (PCA), hierarchical clustering and PERMANOVA is provided in Supplementary Dataset S1.

## 3. Results

### 3.1. Phenotypic Variability Among Ecological Groups

The phenotypic characterization of the forty *L. plantarum* strains revealed substantial variability among isolates originating from different ecological niches. Descriptive statistics for the investigated traits are reported in Table 1.

Differences were observed in enzymatic activity, growth performance, carbohydrate utilization, and acidification capacity. Although considerable strain-to-strain variability was present within each ecological group, distinct trends emerged according to the source of isolation.

Wine-associated strains showed the highest mean β-glucosidase activity (4.68 ± 1.25 nmol pNP), whereas trout isolates exhibited the lowest values (1.53 ± 0.66 nmol pNP). Honeybee and infant isolates displayed intermediate activities. Honeybee-associated strains displayed intermediate values for most traits, while infant-derived isolates showed considerable heterogeneity, reflecting the complexity of the gastrointestinal ecosystem from which they were recovered. Overall, the results indicate that ecological origin contributes to phenotypic variability; however, substantial strain-to-strain heterogeneity within each ecological group suggests that ecological origin alone does not fully explain the diversity observed among isolates.

### 3.2. β-Glucosidase Activity

**Figure 1:**
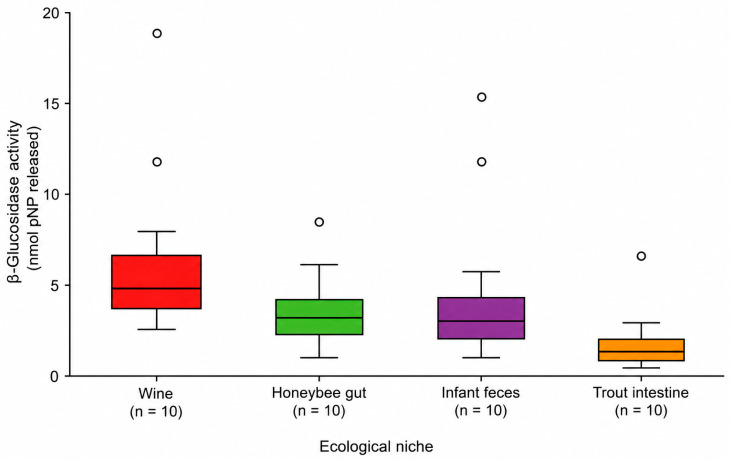
β-Glucosidase activity of *Lactiplantibacillus plantarum* strains isolated from four ecological niches (wine, honeybee gut, trout intestine, and infant feces). Boxplots show enzymatic activity expressed as nmol p-nitrophenol (pNP) per milliliter released during the assay (6 h). Boxes represent the interquartile range, horizontal lines indicate medians, whiskers represent non-outlier values, and circles indicate outliers. Significant differences among ecological groups were detected by the Kruskal–Wallis test (*p* = 0.001). Dunn’s post hoc analysis with Holm correction identified a significant pairwise difference only between wine-associated and trout-associated strains (*p* < 0.05).

β-Glucosidase activity differed significantly among the four ecological groups (Kruskal–Wallis test, *p* = 0.001; Figure 1). Dunn’s post hoc analysis with Holm correction indicated that only wine-associated and trout-associated strains differed significantly, whereas no other pairwise comparisons remained significant after correction for multiple testing (Supplementary Table S3).

Wine-derived strains exhibited the highest enzymatic activity, whereas trout isolates displayed the lowest values. Honeybee- and infant-derived strains showed intermediate activities, although the only statistically supported pairwise difference identified by Dunn’s post hoc test was between wine-associated and trout-associated strains. The higher β-glucosidase activities observed in wine- and honeybee-derived strains may reflect adaptation to environments naturally enriched in plant-derived glycosides and other complex phytochemicals. Conversely, trout-derived isolates generally exhibited lower enzymatic activities, suggesting reduced selective pressure for the utilization of glycosylated substrates in aquatic gastrointestinal environments.

The distribution of β-glucosidase activity revealed a broad phenotypic spectrum within the species, highlighting the presence of strains with markedly different capacities to hydrolyze glycosylated substrates. This enzymatic trait represented one of the most discriminating variables in the dataset and contributed substantially to the differentiation observed in subsequent multivariate analyses.

### 3.3. Global Phenotypic Diversity

**Figure 2:**
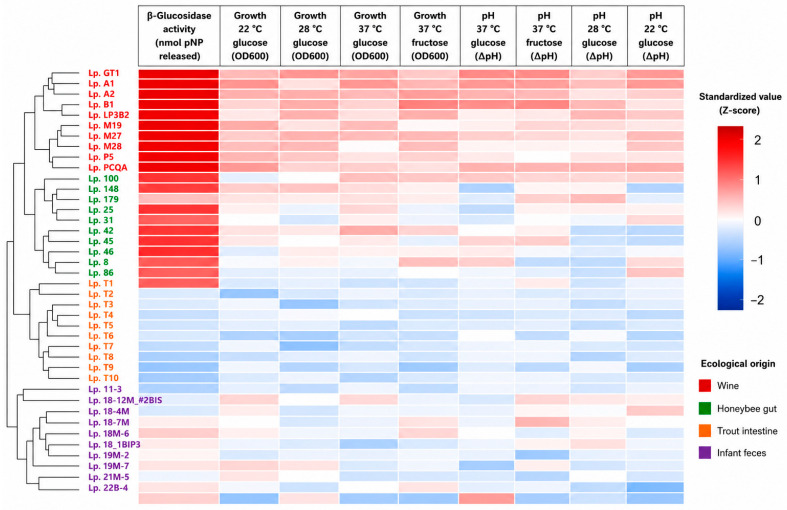
Heatmap of standardized (Z-score) phenotypic traits of 40 *L. plantarum* strains isolated from four ecological niches. Rows represent individual strains and columns represent β-glucosidase activity, growth performance, and acidification traits. Hierarchical clustering was performed using Euclidean distance and Ward’s linkage method. Strain labels are colored according to ecological origin.

The heatmap generated from standardized phenotypic data revealed extensive heterogeneity among the forty strains (Figure 2).

Several traits showed marked variability across isolates, including β-glucosidase activity, growth at different temperatures, fructose utilization, and acidification performance. The heatmap also highlighted phenotypically similar clusters composed of strains sharing common trait combinations. Notably, strains with high β-glucosidase activity tended to cluster together with specific growth and acidification profiles, suggesting the existence of coordinated functional trait combinations.

Although complete separation by ecological origin was not observed, strains from the same habitat often displayed similar phenotypic patterns, suggesting that ecological origin may contribute to the structuring of functional diversity within the species. In particular, wine- and honeybee-associated strains frequently clustered in regions characterized by relatively high β-glucosidase activity and efficient fructose utilization, whereas trout-derived isolates were more often associated with lower enzymatic activity profiles. Nevertheless, the substantial overlap among ecological groups highlights the remarkable phenotypic plasticity that characterizes *L. plantarum*.

### 3.4. Principal Component Analysis

The PCA was performed using β-glucosidase activity, growth performance under different temperature and carbon-source conditions, and acidification traits. The complete strain-level dataset used for the multivariate analyses is reported in Supplementary Dataset S1. The first two principal components explained 74.9% of the total phenotypic variance (PC1 = 58.0%; PC2 = 16.9%). The contribution of each variable to the first two principal components is reported in Table 2.

**Figure 3:**
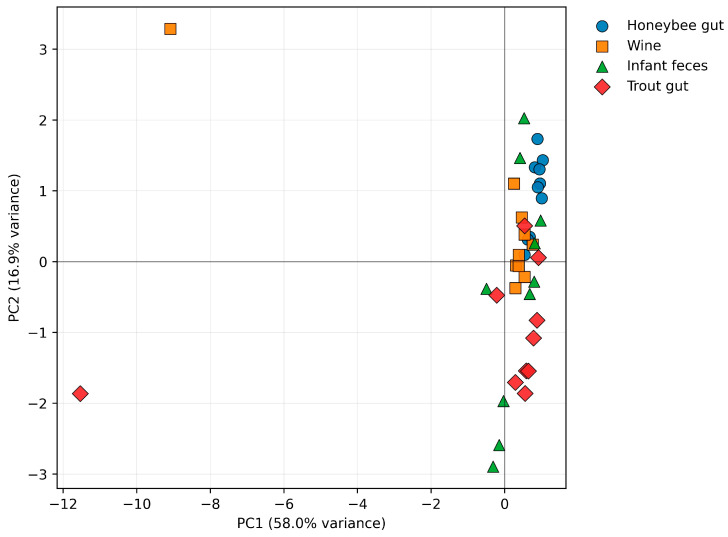
Principal component analysis (PCA) of *L. plantarum* strains based on growth, acidification, and β-glucosidase activity data. Each point represents an individual strain colored according to ecological origin. PC1 and PC2 explain 74.9% of the total phenotypic variance.

PC1 was mainly associated with growth performance and acidification traits, while PC2 was influenced by β-glucosidase activity and differential utilization of fructose relative to glucose (Table 2). PCA revealed weak grouping tendencies according to ecological origin, although extensive overlap among ecological groups was observed. These results indicate that habitat contributes to phenotypic variation but does not result in a clear separation of strains into distinct ecological groups (Figure 3).

The influence of ecological origin on overall phenotypic organization was further supported by PERMANOVA analysis, which revealed a significant effect of habitat on strain phenotypes (*p* = 0.006; Supplementary Table S2). Homogeneity of multivariate dispersions did not differ significantly among ecological groups (PERMDISP, F = 2.183, *p* = 0.107; Supplementary Table S2), indicating that the significant PERMANOVA result was unlikely to be driven primarily by differences in within-group variability. Ecological origin accounted for 15.3% of the total phenotypic variance (R^2^ = 0.153), indicating that habitat is a significant, though not exclusive, determinant of phenotypic diversification.

The remaining 84.7% of the variance was attributable to factors not explained by ecological origin, indicating substantial strain-level heterogeneity within each ecological group. This result suggests that ecological origin contributes to phenotypic structuring, but that individual strain characteristics remain the predominant source of variability within the dataset. A graphical representation of variable contributions to the first two principal components is shown in Supplementary Figure S1. Consistent with the PCA loadings, β-glucosidase activity and differential fructose utilization made the strongest positive contributions to PC2, while growth and acidification traits were primarily associated with PC1 (Supplementary Figure S1).

### 3.5. Cluster Analysis

**Figure 4:**
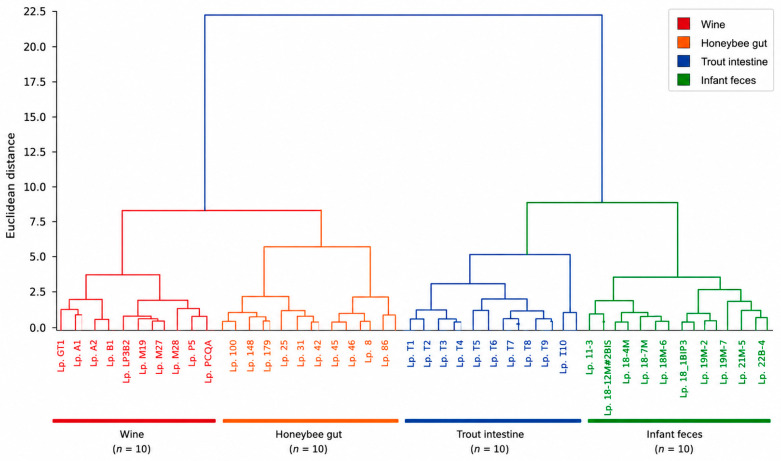
Hierarchical clustering of 40 *L. plantarum* strains based on standardized phenotypic traits. Clustering was performed using Euclidean distance and Ward’s linkage method. Strain labels are colored according to ecological origin.

Hierarchical clustering of the complete phenotypic dataset identified several groups of strains with similar functional characteristics (Figure 4). Although some clusters were enriched with strains from the same habitat, complete separation by ecological origin was not observed, indicating that ecological association contributes to phenotypic diversification while substantial strain-specific variability remains.

The resulting dendrogram confirmed the high level of phenotypic diversity observed in the heatmap and PCA. Clusters were not exclusively associated with a single ecological origin; however, strains from the same habitat occasionally grouped within common branches of the dendrogram, although extensive intermixing among ecological groups remained evident, indicating a partial influence of ecological origin on phenotypic similarity. This observation is consistent with the PERMANOVA results, which identified ecological origin as a significant contributor to phenotypic variation despite extensive overlap among groups.

Consistent with the heatmap and PCA results, β-glucosidase activity contributed to the clustering pattern observed among strains, further supporting its relevance as a niche-associated functional trait.

This pattern suggests that ecological origin helps shape phenotypic profiles, although other factors, including strain-specific adaptation and genomic plasticity, likely play an important role in determining the overall diversity of the species.

Taken together, the clustering results reinforce the view that ecological origin contributes to the organization of phenotypic diversity in *L. plantarum*, while the absence of strict habitat-specific clusters highlights the extensive adaptive flexibility that characterizes this species.

## 4. Discussion

### 4.1. Ecological Origin Contributes to Phenotypic Diversification

The results of this study demonstrate that ecological origin contributes significantly to the phenotypic diversification of *L. plantarum* populations. Although substantial variability was observed among individual strains, several phenotypic traits, including growth performance, acidification capacity, and β-glucosidase activity, showed trends associated with the habitat of isolation (Table 1, Figure 1). These observations support the hypothesis that environmental conditions in different ecological niches may influence the functional characteristics of *L. plantarum* populations.

The effect of habitat on overall phenotypic structure was confirmed by PERMANOVA analysis, which revealed a significant influence of ecological origin on strain phenotypes (*p* = 0.006). This result indicates that ecological origin contributes significantly to the organization of phenotypic diversity and suggests that ecological niche is a relevant factor contributing to intraspecific diversification.

These observations are consistent with recent comparative genomic studies reporting ecological origin-associated differences in carbohydrate metabolism, stress-response mechanisms, and functional gene repertoires among *L. plantarum* strains isolated from diverse habitats [5,10,13,14]. This interpretation is consistent with recent comparative genomic evidence indicating that ecological adaptation in *L. plantarum* is not necessarily reflected by habitat-specific phylogenetic clustering. Cen et al. [12] demonstrated that strains isolated from humans, dairy products, vegetables and *Drosophila* retained distinct repertoires of functional genes despite the absence of clear phylogenetic segregation, suggesting that environmental selection acts primarily on metabolic and adaptive traits rather than on lineage diversification. Similarly, comparative genomic analyses of strains from different ecological niches have revealed habitat-associated differences in carbohydrate metabolism, stress response systems and carbohydrate-active enzymes, supporting the view that ecological pressures contribute to shaping functional diversity while preserving the broad adaptive potential characteristic of the species.

Although statistically significant, ecological origin accounted for only 15.3% of the total phenotypic variance (R^2^ = 0.153). Moreover, homogeneity of multivariate dispersions was not significantly different among ecological groups (PERMDISP, *p* = 0.107), indicating that the significant PERMANOVA result was not primarily attributable to unequal within-group variability. This supports the interpretation that ecological origin contributes to the organization of phenotypic diversity, although its overall effect remains modest relative to strain-level variation. From a biological perspective, this finding is particularly informative. Rather than indicating weak ecological adaptation, the relatively moderate R^2^ value suggests that habitat is one of several interacting factors shaping phenotypic diversity within the species. Indeed, a large proportion of the observed variability remained attributable to strain-specific characteristics, reflecting the remarkable genomic plasticity and adaptive potential that characterize *L. plantarum* [2,7]. Consequently, strains isolated from the same ecological niche may display substantially different phenotypes, while strains from different habitats may converge towards similar functional profiles, as evidenced by the overlap observed in the PCA and hierarchical clustering analyses (Figure 3 and Figure 4). Therefore, the observed phenotypic patterns should be interpreted as ecological origin-associated trends rather than habitat-specific signatures shared uniformly by all strains within a given group.

The ecological niches examined in this study differ significantly in nutrient availability, physicochemical conditions, and selective pressures. Wine-associated environments are characterized by low pH, high concentrations of organic acids, ethanol, and grape-derived phenolic compounds, while the honeybee gut is enriched with nectar- and pollen-derived carbohydrates and phytochemicals. Likewise, the gastrointestinal tracts of trout and pre-weaning infants represent distinct host-associated ecosystems that differ in temperature, dietary composition, and microbial community structure. It is also known that exposure to contrasting environmental conditions may be associated with the distribution of different LAB exhibiting specific metabolic traits [30,31].

Despite their different ecological origins, all strain groups showed robust growth across the temperature range tested, indicating that thermal adaptability remains a conserved characteristic of *L. plantarum*. This observation aligns with the nomadic lifestyle of the species and its capacity to colonize diverse environments without strong temperature specialization [6,9].

Although growth performance varied quantitatively among ecological groups, particularly at 22 °C and 28 °C, all strains retained the ability to proliferate efficiently across the entire temperature range tested. This suggests that temperature is not a primary driver of phenotypic differentiation within the species. A similar pattern was observed for acidification capacity. Although minor differences among ecological groups were detected, all strains efficiently reduced the pH of the growth medium, indicating that acid production remains a conserved functional trait across the species. This suggests that fermentative metabolism is a core ecological and physiological characteristic of *L. plantarum*, regardless of habitat of origin.

Therefore, the phenotypic differences observed among ecological groups are consistent with the hypothesis that habitat-specific selective pressures contribute to shaping strain functionality over time, although confirmation of the underlying adaptive mechanisms will require integrated genomic and transcriptomic investigations.

Taken together, these findings support the view that ecological origin contributes to the functional diversification of *L. plantarum* populations, while also highlighting the extraordinary phenotypic heterogeneity that characterizes the species. Habitat appears to influence the distribution of functional traits but does not impose strict ecological specialization, suggesting a dynamic balance between environmental adaptation and the intrinsic versatility of this highly adaptable bacterium.

### 4.2. Phenotypic Diversity in a Nomadic Species

Although ecological origin significantly influenced the phenotypic organization of the strain collection, neither principal component analysis nor hierarchical clustering revealed a complete separation of strains by habitat of isolation. The PCA therefore should not be interpreted as evidence of distinct ecological clusters, but rather as indicating weak grouping tendencies superimposed on extensive strain-level variability (Figure 3 and Figure 4). Instead, substantial overlap among ecological groups was observed, with several strains from different ecological origins clustering together despite their distinct origins. This finding highlights the remarkable functional versatility of *L. plantarum* and supports the concept of a species characterized by a predominantly nomadic lifestyle [32].

The nomadic nature of *L. plantarum* has been extensively documented through comparative genomic studies, which have revealed an open and highly flexible genome enriched in genes involved in carbohydrate utilization, environmental adaptation, and stress response mechanisms [2,7]. Unlike highly specialized microorganisms that evolve in association with a restricted ecological niche, *L. plantarum* appears capable of colonizing and persisting in a broad spectrum of environments, ranging from plant-derived fermentations to animal gastrointestinal tracts. This ecological versatility is reflected in its extensive phenotypic heterogeneity and the absence of strict habitat-dependent clustering.

The heatmap analysis further supported this interpretation by revealing considerable variability among strains, even within the same ecological group (Figure 2). In several cases, strains isolated from identical habitats exhibited markedly different phenotypic profiles, while strains from different niches shared similar combinations of growth, acidification, and metabolic traits. This pattern suggests that phenotypic diversification within *L. plantarum* cannot be explained solely by ecological origin but likely reflects the interaction of multiple factors, including genomic plasticity, environmental influences, and strain-specific functional characteristics. This interpretation is further supported by recent genomic evidence showing that *L. plantarum* strains frequently share conserved functional capacities while simultaneously displaying extensive strain-level variability in accessory genes and metabolic traits, highlighting the importance of intraspecific diversification in shaping ecological and technological properties [9].

The ability to efficiently utilize fructose was widely conserved among strains, with all ecological groups showing comparable or slightly improved growth in fructose-containing medium compared to glucose. This finding is consistent with the ecology of several habitats examined in this study. In particular, grape must and grape-derived products are naturally rich in fructose, while nectar and pollen consumed by honeybees also contain high concentrations of simple sugars, including fructose. Efficient utilization of this carbohydrate may therefore represent an advantageous metabolic trait in plant-associated environments. Notably, variation in the magnitude of the fructose response contributed substantially to PC2, suggesting that differential carbohydrate utilization is an additional source of phenotypic diversification within the species.

From a biochemical perspective, fructose utilization is particularly relevant because this sugar represents a major carbohydrate component of nectar, honey, and grape-derived substrates. Unlike glucose, fructose may be transported and metabolized through distinct regulatory pathways, and efficient fructose utilization can provide a competitive advantage in environments where fructose is abundant. Consequently, the enhanced fructose utilization observed in honeybee- and wine-associated strains may reflect functional characteristics associated with persistence and metabolic activity in sugar-rich ecological niches. In contrast, glucose is a ubiquitous carbon source available across a wide range of environments and is therefore less informative as a marker of ecological origin-associated phenotypic variation.

The PCA loadings (Table 2) indicated that growth performance and acidification parameters contributed most strongly to PC1, while β-glucosidase activity and differential fructose utilization contributed substantially to PC2. Notably, β-glucosidase activity emerged as one of the principal metabolic traits associated with the second component, supporting its relevance as a functional marker of phenotypic diversification within the species.

Although ecological origin explained a significant proportion of the observed phenotypic variation, the PERMANOVA results also indicated that most of the variability remained within ecological groups. This finding suggests that strain-specific differences contribute substantially to the overall phenotypic diversity of *L. plantarum* and highlights the importance of considering both ecological origin and intra-group heterogeneity when interpreting functional variation within the species. Although habitat exerts a measurable influence on the distribution of functional traits, most phenotypic variation remains associated with strain-specific characteristics. Similar conclusions have been reported in previous genomic and phenotypic investigations, where ecological origin-dependent trends were observed, but complete niche specialization was rarely detected [7,10,11].

Experimental studies performed on plant-associated populations further support this interpretation. Yu et al. [14] reported that *L. plantarum* strains isolated from olive, tomato, cereal and cactus fermentations exhibited niche-related differences in carbohydrate utilization and stress tolerance despite belonging to the same species. These observations suggest that ecological adaptation in *L. plantarum* frequently occurs through modulation of functional traits rather than through the emergence of highly specialized ecotypes.

Consequently, *L. plantarum* can be regarded as a species occupying an intermediate position between ecological specialization and ecological generalism, combining broad environmental adaptability with the expression of ecological origin-associated phenotypic traits.

The coexistence of habitat-associated signatures and extensive strain-level variability may represent one of the key ecological strategies underlying the success of *L. plantarum*. Rather than evolving into distinct habitat-specific lineages, populations appear to maintain a large reservoir of phenotypic diversity that can be selectively favored under different environmental conditions. This adaptive strategy likely contributes to the widespread distribution of the species and its ability to thrive in ecosystems characterized by highly variable nutritional resources and physicochemical constraints.

### 4.3. β-Glucosidase as an Ecological Origin-Associated Functional Trait

Among the phenotypic traits investigated, β-glucosidase activity emerged as one of the most discriminating characteristics among the ecological groups examined and was the clearest functional trait associated with the habitat of isolation (Table 1; Figure 1). Significant overall differences in β-glucosidase activity were detected among ecological groups. However, Dunn’s post hoc analysis indicated that only wine-associated and trout-associated strains differed significantly after correction for multiple testing. Honeybee- and infant-derived strains displayed intermediate activity levels, although these differences were not statistically supported in pairwise comparisons.

These findings suggest that β-glucosidase activity may serve as a useful phenotypic marker associated with ecological origin. The contribution of β-glucosidase activity to PC2 in the multivariate analysis (Table 2) further supports the importance of this trait in structuring phenotypic diversity among strains.

β-Glucosidases catalyze the hydrolysis of β-glycosidic bonds found in a wide range of plant-derived compounds, releasing glucose and biologically active aglycones.

Beyond simple carbohydrate acquisition, β-glucosidases play a central role in the ecological interaction between *L. plantarum* and plant-derived substrates. These enzymes are involved in the release of volatile aroma compounds from glycosylated precursors, the conversion of phenolic glycosides into more bioavailable aglycones, the debittering of olive products through oleuropein hydrolysis, and the transformation of various phytochemicals occurring in fermented plant foods. Consequently, β-glucosidase activity may provide both nutritional and ecological advantages in environments enriched in plant secondary metabolites.

In food fermentations, these enzymes play a key role in liberating volatile aroma compounds from non-volatile glycosylated precursors, thus contributing to flavor development and sensory quality [22,23]. Furthermore, the hydrolysis of glycosylated substrates may increase the availability of carbon sources and bioactive molecules, providing a potential ecological advantage in environments rich in plant-derived metabolites [24].

The elevated β-glucosidase activity observed in wine-associated strains (Table 1) may reflect adaptation to the specific characteristics of the wine ecosystem. Grapes and grape-derived products contain numerous glycosylated aroma precursors and phenolic compounds that serve as substrates for microbial glycosidases. Consequently, strains with higher β-glucosidase activity may benefit from an expanded metabolic repertoire and enhanced ecological efficiency in this environment. Similar findings have been reported for wine-associated *L. plantarum* strains, which frequently display enzymatic activities involved in aroma release and the transformation of grape-derived compounds during fermentation [23,33]. The highest average enzymatic activity recorded among wine isolates in this study further supports the hypothesis that this trait may be favored in environments characterized by an abundance of plant-derived glycosides. In such habitats, β-glucosidase-mediated hydrolysis may increase access to fermentable sugars and bioactive aglycones, providing a potential ecological advantage and favoring the persistence of strains exhibiting elevated enzymatic activity.

Collectively, these observations support the hypothesis that β-glucosidase activity represents one of the most informative phenotypic markers associated with ecological origin in plant-associated environments. Recent studies have highlighted that spontaneous plant fermentations constitute reservoirs of highly diverse *L. plantarum* populations enriched in carbohydrate-active enzymes and metabolic functions involved in the degradation of complex plant substrates. In this context, elevated β-glucosidase activity may represent part of a broader adaptive strategy enabling efficient exploitation of glycosylated compounds naturally present in fruits, nectar, pollen and fermented plant matrices [14,25,33,34]. Notably, honeybee-derived strains also exhibited relatively high β-glucosidase activities (Table 1). Although honeybee-derived strains exhibited relatively high mean β-glucosidase activities, the differences relative to the other ecological groups were not statistically significant after correction for multiple comparisons and should therefore be interpreted as descriptive trends.

The honeybee gastrointestinal tract is naturally exposed to nectar, pollen, and bee-derived products, all of which are rich in glycosylated compounds, flavonoids, and plant secondary metabolites. Previous investigations have shown that *L. plantarum* strains associated with honeybees possess various functional traits linked to carbohydrate metabolism and adaptation to phytochemical-rich environments [21]. The enzymatic activities observed in this study are therefore consistent with the nutritional characteristics of this ecological niche and may contribute to the efficient utilization of plant-derived substrates.

In contrast, trout-derived isolates showed the lowest β-glucosidase activities among all ecological groups (Table 1). Although the mechanisms underlying this observation remain speculative, aquatic gastrointestinal environments generally contain fewer plant-derived glycosides than terrestrial plant-associated habitats. Additionally, rainbow trout is predominantly carnivorous, and its gastrointestinal environment is typically exposed to lower amounts of plant-derived glycosides than environments associated with fruits, nectar, or pollen. Consequently, the selective pressure to maintain high β-glucosidase activity may be reduced, contributing to the lower enzymatic activities observed among trout-derived isolates. While further studies are needed to confirm this hypothesis, the observed pattern suggests that substrate availability may play an important role in shaping the distribution of β-glucosidase activity among *L. plantarum* populations.

Despite clear habitat-associated trends, substantial variability remained among strains within the same ecological group (Figure 1). This indicates that β-glucosidase activity is not determined solely by ecological origin but is also influenced by strain-specific genetic and regulatory factors. Previous studies have reported considerable intraspecific variation in β-glucosidase production among *L. plantarum* isolates, supporting the existence of multiple adaptive strategies within the species [22,24,29]. Such variability likely reflects the combined effects of genomic plasticity, local adaptation, and differential regulation of carbohydrate-active enzymes.

Taken together, these findings indicate that β-glucosidase activity is a valuable functional marker for investigating ecological adaptation in *L. plantarum*. The significant differences observed among ecological groups suggest that habitat-associated selective pressures contribute to shaping the distribution of this enzymatic trait. At the same time, the persistence of substantial strain-level variability reinforces the view that adaptive flexibility and phenotypic diversification remain defining characteristics of this remarkably versatile bacterial species.

### 4.4. Technological Implications

Beyond their ecological significance, the phenotypic differences observed among *L. plantarum* strains may have important technological implications. The considerable variability in growth performance, acidification capacity, and β-glucosidase activity highlights the existence of functionally distinct populations that could be exploited for specific industrial and biotechnological applications. This diversity is a valuable resource for selecting strains with desirable technological traits, rather than relying solely on species-level identification. Among the traits investigated, β-glucosidase activity is of particular interest because of its direct impact on the transformation of glycosylated compounds during food fermentations. Strains with elevated β-glucosidase activity may contribute to the release of aroma-active molecules and bioactive aglycones, enhancing the sensory complexity and functional value of fermented products [22,23,24].

β-Glucosidase activity is known to hydrolyze a wide range of glycosylated precursors naturally occurring in plant-derived foods. In wine and grape products, β-glucosidases may release volatile aroma compounds such as linalool, geraniol, nerol and benzyl alcohol from their corresponding glycosidic precursors, thereby enhancing aroma complexity and varietal character [15,16,23]. In addition, these enzymes contribute to the hydrolysis of phenolic glycosides and flavonoid conjugates, generating bioactive aglycones with increased bioavailability and biological activity. Similar mechanisms have been reported for the transformation of oleuropein derivatives in olives and for the release of antioxidant phenolic compounds during plant fermentations [15,17].

In this context, the higher enzymatic activities observed in wine-associated isolates (Table 1; Figure 1) suggest that these strains may be promising candidates for applications involving aroma enhancement and flavor development during fermentation processes. Differences in growth and acidification performance may also be technologically relevant. Rapid growth and efficient acidification are key characteristics of starter cultures, as they contribute to microbial dominance, product stability, and inhibition of undesirable microorganisms. The variability observed among strains under different incubation temperatures and carbon sources (Table 1) indicates that individual isolates may be particularly suited to specific fermentation conditions or food matrices. Such functional diversity could facilitate the development of tailored starter cultures adapted to distinct technological requirements. The results of this study further emphasize the importance of strain-level selection in *L. plantarum*. Although strains belong to the same species, they may differ substantially in metabolic capabilities and functional performance. Consequently, the identification and characterization of strains possessing desirable phenotypic traits remain essential steps for the development of innovative food fermentations, functional foods and other biotechnological applications. The combination of ecological screening and phenotypic characterization may therefore represent an effective strategy for discovering strains with enhanced technological potential.

### 4.5. Limitations and Future Perspectives

Although the present study provides evidence that ecological origin contributes to phenotypic diversification in *L. plantarum*, some limitations should be acknowledged. First, the investigation relied exclusively on phenotypic characterization, so the genetic determinants underlying the observed differences could not be directly assessed. While growth performance, acidification capacity, and β-glucosidase activity showed significant variability among ecological groups, the molecular mechanisms responsible for these phenotypic traits remain largely unexplored. Furthermore, although forty strains from four distinct ecological niches were included, the strain collection may not fully represent the diversity of *L. plantarum* populations in natural and fermented environments. Additional isolates from a broader range of habitats would provide a more comprehensive understanding of the relationship between ecological origin and functional diversification. Likewise, the phenotypic traits evaluated in this study represent only a subset of the characteristics potentially involved in ecological adaptation. Other functions, such as stress tolerance, antimicrobial activity, biofilm formation, and host-interaction capabilities, may also contribute significantly to niche-associated differentiation. In addition, β-glucosidase activity was evaluated under a single incubation temperature (37 °C). Although this condition was selected because it supported the highest overall growth performance of most strains, isolates originating from ectothermic hosts such as trout may exhibit different enzymatic responses under lower temperature conditions. Future studies should therefore investigate the temperature dependence of β-glucosidase activity to better understand the ecological significance of this trait across different habitats.

Future investigations should integrate phenotypic analyses with whole-genome sequencing and comparative genomic approaches to identify the genetic basis of the observed functional variability. In particular, the characterization of the genes involved in carbohydrate metabolism and β-glucosidase production could provide valuable insights into the evolutionary processes linking ecological adaptation and metabolic specialization. Transcriptomic and proteomic analyses may further clarify the regulatory mechanisms controlling the expression of key functional traits under different environmental conditions.

Despite these limitations, this study demonstrates that populations of *L. plantarum* isolated from ecologically distinct habitats retain measurable functional signatures while preserving the extensive phenotypic heterogeneity characteristic of the species. These findings support the view that ecological adaptation and genomic versatility together shape intraspecific biodiversity and highlight the importance of strain-level characterization for both ecological investigations and biotechnological applications.

## 5. Conclusions

This study demonstrates that ecological origin significantly contributes to the phenotypic diversification of *L. plantarum* populations. Strains isolated from wine, honeybee gut, trout intestine, and infant feces showed distinct functional profiles in growth performance, acidification capacity, and β-glucosidase activity, indicating that ecological origin may contribute to the distribution of phenotypic traits.

The significant effect of ecological origin detected by PERMANOVA supports the existence of measurable ecological origin-associated phenotypic signatures. However, ecological origin accounted for only a limited proportion of the total phenotypic variance, indicating that strain-level variability is the dominant component of diversity within the species.

The extensive overlap observed among ecological groups in the multivariate analyses highlights the remarkable phenotypic heterogeneity and adaptive versatility that characterize *L. plantarum*. The coexistence of habitat-related trends and substantial strain-level variability is consistent with the concept of a nomadic species capable of thriving across a wide range of environments while retaining the capacity to express ecological origin-associated phenotypic traits.

Among the traits investigated, β-glucosidase activity emerged as the most informative marker of ecological origin-associated phenotypic differentiation. The higher β-glucosidase activity observed in wine-associated strains, together with its strong contribution to PC2, suggests that this enzymatic function may represent an important ecological origin-associated trait and a potentially relevant determinant of technological performance. Overall, the results indicate that ecological origin contributes to phenotypic diversification while phenotypic plasticity remains a defining characteristic of *L. plantarum*. These findings enhance understanding of the ecological biology of this species and provide a valuable basis for selecting strains with desirable functional and technological properties for food, probiotic, and biotechnological applications. Future studies integrating phenotypic characterization with genomic analyses will further clarify the molecular basis of ecological origin-associated functional diversification in *L. plantarum*.

## Figures and Tables

**Table 1:** Mean values (±standard deviation) of the phenotypic traits evaluated in *L. plantarum* strains isolated from four ecological niches (honeybee gut, infant feces, wine, and trout intestine).

Phenotypic Trait	Honeybee Gut	Infant Feces	Wine	Trout Intestine
β-Glucosidase activity (nmol pNP mL^−1^ in 6 h)	3.406 ± 1.769	3.198 ± 2.085	4.681 ± 1.251	1.534 ± 0.656
Growth at 22 °C (OD_600_)	1.529 ± 0.127	0.974 ± 0.322	0.779 ± 0.114	0.887 ± 0.327
Growth at 28 °C (OD_600_)	2.759 ± 0.063	2.595 ± 0.300	2.088 ± 0.199	2.373 ± 0.859
Growth at 37 °C (OD_600_)	2.813 ± 0.014	2.773 ± 0.011	2.536 ± 0.870	2.485 ± 0.835
Growth on fructose at 37 °C (OD_600_)	2.817 ± 0.013	2.653 ± 0.182	2.521 ± 0.862	2.372 ± 0.821
Final pH (37 °C, glucose)	3.691 ± 0.057	3.669 ± 0.103	3.854 ± 0.576	3.707 ± 0.408
Final pH (37 °C, fructose)	3.835 ± 0.058	3.767 ± 0.075	3.962 ± 0.514	3.797 ± 0.376
Final pH (28 °C, glucose)	3.890 ± 0.021	3.916 ± 0.103	3.823 ± 0.018	4.065 ± 0.602
Final pH (22 °C, glucose)	4.134 ± 0.047	4.144 ± 0.122	4.015 ± 0.596	4.286 ± 0.588

Values are reported as mean ± standard deviation of three independent replicates. OD_600_ values refer to bacterial growth in MRS supplemented with glucose or fructose. Acidification capacity is expressed as final pH values. β-Glucosidase activity was expressed as nmol of p-nitrophenol (pNP) released per milliliter of reaction mixture at the end of the experiment (nmol pNP mL^−1^ in 6 h).

**Table 2:** Loadings of phenotypic variables on the first two principal components (PC1 and PC2) obtained from principal component analysis (PCA). Variables with higher absolute loading values contributed most strongly to the multivariate structure observed among *L. plantarum* strains.

Variable	PC1 Loading	PC2 Loading
β-Glucosidase activity	0.056	0.471
Growth at 22 °C (glucose)	0.232	0.374
Growth at 28 °C (glucose)	0.298	0.267
Growth at 37 °C (glucose)	0.404	−0.107
Growth at 37 °C (fructose)	0.404	0.006
ΔOD fructose − glucose (37 °C)	0.018	0.603
Acidification at 22 °C (glucose)	0.386	−0.058
Acidification at 28 °C (glucose)	0.313	0.202
Acidification at 37 °C (glucose)	0.374	−0.274
Acidification at 37 °C (fructose)	0.375	−0.270

## Data Availability

The raw data supporting the conclusions of this article have been provided as Supplementary Materials.

## Supplementary Materials

The following supporting information can be downloaded at: https://www.mdpi.com/article/10.3390/cimb48070683/s1.
