# Supplementary material for: Phenotypic Biodiversity and Niche-Associated Functional Traits in Lactiplantibacillus plantarum
Source: Curr Issues Mol Biol. 2026 Jul 2;48(7):683. doi: 10.3390/cimb48070683 (PMC13406659; doi:10.3390/cimb48070683)
Supplement: Supplementary file 1 [file cimb-48-00683-s001.zip › cimb-4395474-supplementary materials.pdf]

Supplementary Table S1. Origin and molecular identification of the *Lactiplantibacillus plantarum* strains included in the study. Wine-associated strains were previously described by [25] and [33], honeybee-derived strains by [26], trout isolates by [27], whereas infant-derived strains originated from a research project approved by the Bioethical Committee of the University of Molise (Protocol No. 21779, 09/05/2024).

Strain	Species	Ecological origin	Original reference (year of isolation)	Identification method	GenBank accession number
Lp. GT1	*Lactiplantibacillus plantarum*	Wine	[25,33]	16S rRNA gene sequencing	OR234805
Lp. A1	*Lactiplantibacillus plantarum*	Wine	[25,33]	16S rRNA gene sequencing	PP379893
Lp. A2	*Lactiplantibacillus plantarum*	Wine	[25,33]	16S rRNA gene sequencing	OR625469
Lp. B1	*Lactiplantibacillus plantarum*	Wine	[25,33]	16S rRNA gene sequencing	OR625466
Lp. LP3B2	*Lactiplantibacillus plantarum*	Wine	[25,33]	16S rRNA gene sequencing	OR234806
Lp. M19	*Lactiplantibacillus plantarum*	Wine	[25,33]	16S rRNA gene sequencing	OR625467
Lp. M27	*Lactiplantibacillus plantarum*	Wine	[25,33]	16S rRNA gene sequencing	PP379894
Lp. M28	*Lactiplantibacillus plantarum*	Wine	[25,33]	16S rRNA gene sequencing	OR625465
Lp. P5	*Lactiplantibacillus plantarum*	Wine	[25,33]	16S rRNA gene sequencing	OR234807
Lp. PCQA	*Lactiplantibacillus plantarum*	Wine	[25,33]	16S rRNA gene sequencing	OR625472
Lp. 100	*Lactiplantibacillus plantarum*	Honeybee gut	[26]	16S rRNA gene sequencing	OM033655
Lp. 148	*Lactiplantibacillus plantarum*	Honeybee gut	[26]	16S rRNA gene sequencing	OM038096

Lp. 179	*Lactiplantibacillus plantarum*	Honeybee gut	[26]	16S rRNA gene sequencing	OM038097
Lp. 25	*Lactiplantibacillus plantarum*	Honeybee gut	[26]	16S rRNA gene sequencing	PP463062
Lp. 31	*Lactiplantibacillus plantarum*	Honeybee gut	[26]	16S rRNA gene sequencing	OM038094
Lp. 42	*Lactiplantibacillus plantarum*	Honeybee gut	[26]	16S rRNA gene sequencing	OM038095
Lp. 45	*Lactiplantibacillus plantarum*	Honeybee gut	[26]	16S rRNA gene sequencing	OP648179
Lp. 46	*Lactiplantibacillus plantarum*	Honeybee gut	[26]	16S rRNA gene sequencing	OP415393
Lp. 8	*Lactiplantibacillus plantarum*	Honeybee gut	[26]	16S rRNA gene sequencing	OM033651
Lp. 86	*Lactiplantibacillus plantarum*	Honeybee gut	[26]	16S rRNA gene sequencing	OM033653
Lp. T1	*Lactiplantibacillus plantarum*	Trout intestine	[27]	16S rRNA gene sequencing	PP400704
Lp. T2	*Lactiplantibacillus plantarum*	Trout intestine	[27]	16S rRNA gene sequencing	PP400705
Lp. T3	*Lactiplantibacillus plantarum*	Trout intestine	[27]	16S rRNA gene sequencing	PP400706
Lp. T4	*Lactiplantibacillus plantarum*	Trout intestine	[27]	16S rRNA gene sequencing	PP400707
Lp. T5	*Lactiplantibacillus plantarum*	Trout intestine	[27]	16S rRNA gene sequencing	PP400708
Lp. T6	*Lactiplantibacillus plantarum*	Trout intestine	[27]	16S rRNA gene sequencing	PP400709
Lp. T7	*Lactiplantibacillus plantarum*	Trout intestine	[27]	16S rRNA gene sequencing	PP400710

Lp. T8	*Lactiplantibacillus plantarum*	Trout intestine	[27]	16S rRNA gene sequencing	PP400711
Lp. T9	*Lactiplantibacillus plantarum*	Trout intestine	[27]	16S rRNA gene sequencing	PP400712
Lp. T10	*Lactiplantibacillus plantarum*	Trout intestine	[27]	16S rRNA gene sequencing	PP400713
Lp. 11-3	*Lactiplantibacillus plantarum*	Infant feces	Protocol No. 21779 (09/05/2024)	16S rRNA gene sequencing	OQ452920
Lp. 18-12M_#2BIS	*Lactiplantibacillus plantarum*	Infant feces	Protocol No. 21779 (09/05/2024)	16S rRNA gene sequencing	OR234815
Lp. 18-4M	*Lactiplantibacillus plantarum*	Infant feces	Protocol No. 21779 (09/05/2024)	16S rRNA gene sequencing	OR234793
Lp. 18-7M	*Lactiplantibacillus plantarum*	Infant feces	Protocol No. 21779 (09/05/2024)	16S rRNA gene sequencing	OR625464
Lp. 18M-6	*Lactiplantibacillus plantarum*	Infant feces	Protocol No. 21779 (09/05/2024)	16S rRNA gene sequencing	OP648182
Lp. 18_1BIP3	*Lactiplantibacillus plantarum*	Infant feces	Protocol No. 21779 (09/05/2024)	16S rRNA gene sequencing	OR234809
Lp. 19M-2	*Lactiplantibacillus plantarum*	Infant feces	Protocol No. 21779 (09/05/2024)	16S rRNA gene sequencing	OP648180
Lp. 19M-7	*Lactiplantibacillus plantarum*	Infant feces	Protocol No. 21779 (09/05/2024)	16S rRNA gene sequencing	OP648183
Lp. 21M-5	*Lactiplantibacillus plantarum*	Infant feces	Protocol No. 21779 (09/05/2024)	16S rRNA gene sequencing	OP415392
Lp. 22B-4	*Lactiplantibacillus plantarum*	Infant feces	Protocol No. 21779 (09/05/2024)	16S rRNA gene sequencing	OR625462

Supplementary Table S2. PERMANOVA and PERMDISP analyses evaluating the effect of ecological origin on the overall phenotypic structure of Lactiplantibacillus plantarum strains.

Analysis	Statistic	p-value
PERMANOVA	$R^2 = 0.153$	0.006
PERMDISP	F = 2.183	0.107

PERMANOVA was performed on a Euclidean distance matrix calculated from the standardized phenotypic dataset using 999 permutations. PERMDISP was used to evaluate homogeneity of multivariate dispersions among ecological groups. No significant differences in dispersion were detected ($p > 0.05$).

Supplementary Table S3. Results of Dunn's post hoc multiple-comparison test with Holm correction applied to β-glucosidase activity among *Lactiplantibacillus plantarum* strains from different ecological origins following a significant Kruskal–Wallis test. Z statistics, Holm-adjusted p-values, and significance levels are reported for all pairwise comparisons. Statistical significance was accepted at $p < 0.05$. Abbreviations: ns, not significant (adjusted $p \geq 0.05$); *, significant difference (adjusted $p < 0.05$).

Comparison	Z statistic	Adjusted p-value (Holm)	Significance
Wine vs Trout	3.998	0.000384	*
Honeybee vs Trout	2.372	0.088512	ns
Infant vs Trout	2.123	0.134972	ns
Wine vs Infant	1.874	0.182595	ns
Wine vs Honeybee	1.626	0.207976	ns
Honeybee vs Infant	0.249	0.803628	ns

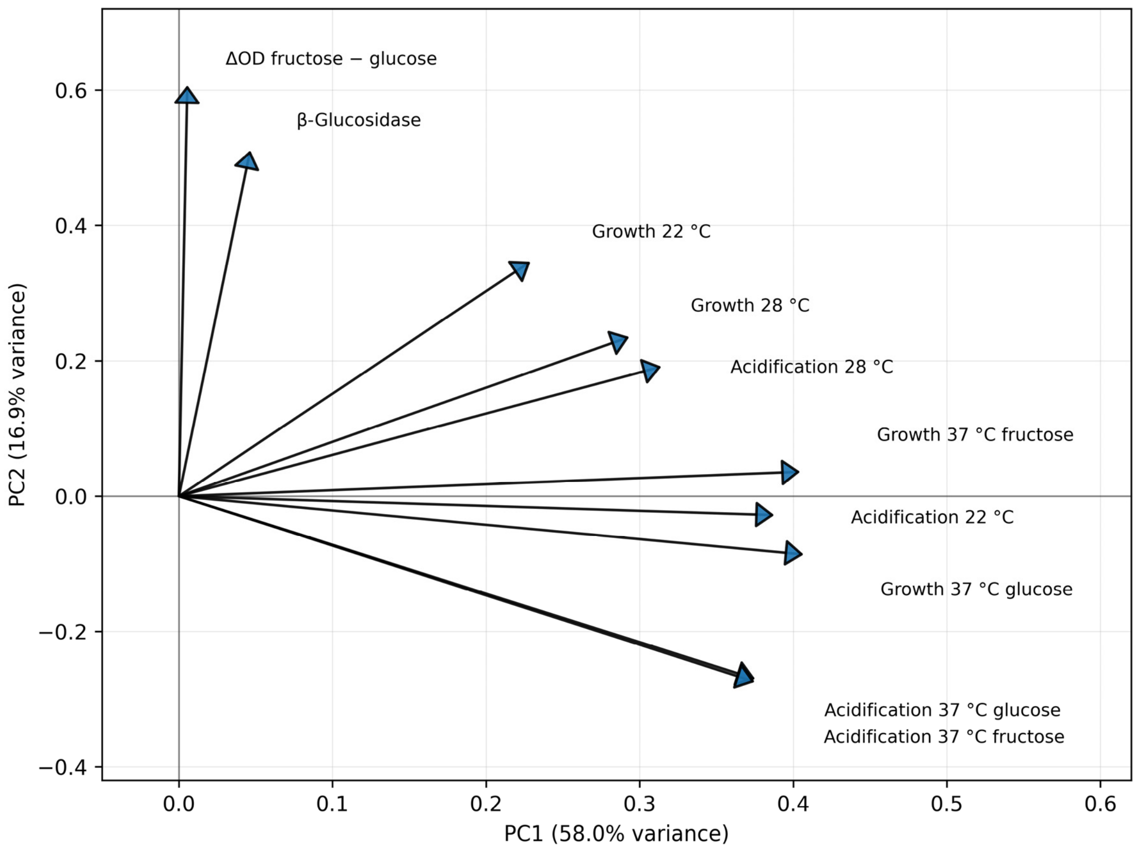

Supplementary Figure S1. Variable loadings on PC1 and PC2.
